# *UMOD* as a susceptibility gene for end-stage renal disease

**DOI:** 10.1186/1471-2350-13-78

**Published:** 2012-09-05

**Authors:** Anna Reznichenko, Carsten A Böger, Harold Snieder, Jacob van den Born, Martin H de Borst, Jeffrey Damman, Marcory CRF van Dijk, Harry van Goor, Bouke G Hepkema, Jan-Luuk Hillebrands, Henri GD Leuvenink, Jan Niesing, Stephan JL Bakker, Marc Seelen, Gerjan Navis

**Affiliations:** 1 Department of Internal Medicine, Division of Nephrology, University Medical Center Groningen, A. Deusinglaan 1, Groningen, 9713AV, the Netherlands; 2 Department of Internal Medicine II, University Medical Center Regensburg, Regensburg, Germany; 3 Department of Epidemiology, Unit of Genetic Epidemiology & Bioinformatics, University Medical Center Groningen, University of Groningen, Groningen, the Netherlands; 4 Department of Surgery, University Medical Center Groningen, University of Groningen, Groningen, the Netherlands; 5 Department of Pathology and Medical Biology, University Medical Center Groningen, University of Groningen, Groningen, the Netherlands; 6 Department of Laboratory Medicine, University Medical Center Groningen, University of Groningen, Groningen, the Netherlands

**Keywords:** UMOD, Uromodulin, Polymorphisms, SNP, End-stage renal disease, Kidney transplantation

## Abstract

**Background:**

In recent genetic association studies, common variants including rs12917707 in the *UMOD* locus have shown strong evidence of association with eGFR, prevalent and incident chronic kidney disease and uromodulin urinary concentration in general population cohorts. The association of rs12917707 with end-stage renal disease (ESRD) in a recent case-control study was only nominally significant.

**Methods:**

To investigate whether rs12917707 associates with ESRD, graft failure (GF) and urinary uromodulin levels in an independent cohort, we genotyped 1142 ESRD patients receiving a renal transplantation and 1184 kidney donors as controls. After transplantation, 1066 renal transplant recipients were followed up for GF. Urinary uromodulin concentration was measured at median [IQR] 4.2 [2.2-6.1] yrs after kidney transplantation.

**Results:**

The rs12917707 minor allele showed association with lower risk of ESRD (OR 0.89 [0.76-1.03], *p* = 0.04) consistent in effect size and direction with the previous report (Böger *et al*, PLoS Genet 2011). Meta-analysis of these findings showed significant association of rs12917707 with ESRD (OR 0.91 [0.85-98], *p* = 0.008). In contrast, rs12917707 was not associated with incidence of GF. Urinary uromodulin concentration was lower in recipients-carriers of the donor rs12917707 minor allele as compared to non-carriers, again consistent with previous observations in general population cohorts.

**Conclusions:**

Our study thus corroborates earlier evidence and independently confirms the association between *UMOD* and ESRD.

## Background

Chronic kidney disease (CKD) is a significant and increasing global challenge for public health. It affects ≈ 10% of the general population in industrialized nations, incurring high morbidity and mortality, and posing a substantial financial burden to the health care systems [1-3]. It is a complex, multifactorial disorder with an important genetic component. Identification of the genetic variants involved in its susceptibility and progression to end-stage renal disease (ESRD) will improve our understanding of biological mechanisms underlying renal function and will ultimately lead to development of novel tools for diagnosis, prevention, prediction and treatment [4-6].

Recent advances in genome-wide association (GWA) studies of kidney disease resulted in discovery of several genes. Among them a prominent place is taken by *UMOD* which has been reproducibly identified in multiple cohorts as one of the top loci associated with renal function parameters [7-10]. Several GWA studies highlighted a region upstream from the *UMOD* gene containing rs12917707 and several other SNPs in high linkage disequilibrium (LD). The mentioned LD block was repeatedly shown to be associated with prevalent and incident CKD, and also uromodulin urinary concentration. All the studies showed a consistent trend of association of the rs12917707 minor allele with lower risk of CKD [7,11-15], and the minor alleles of SNPs in perfect LD with rs12917707, rs4293393 and rs13333226, were associated with lower urinary uromodulin levels [11,15].

A recent study examined the role of rs12917707 genotype in risk for a more severe renal phenotype, ESRD, with the minor allele again showing a protective effect: OR [95% CI] 0.92 [0.86-1.0] [14]. However, the level of statistical significance was only nominal (*p* = 0.04), warranting further investigation to confirm the association of the *UMOD* variants with kidney damage phenotypes.

We thus analyzed the association of rs12917707 with ESRD and with graft failure (GF) after kidney transplantation, and investigated the effect of rs12917707 genotype on urinary uromodulin levels.

**Figure 1:**
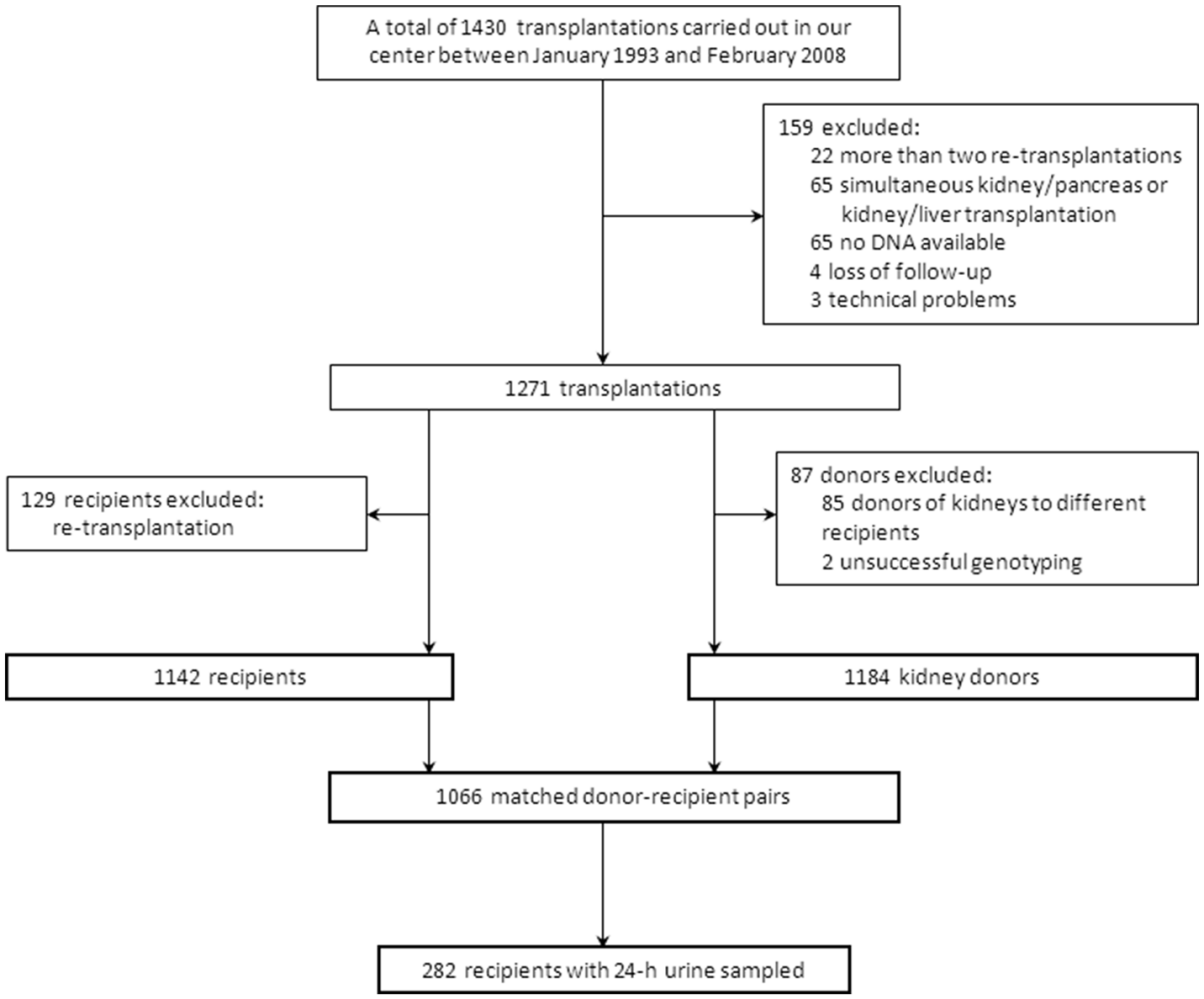
A flowchart of the study participants selection.

First, we performed a case-control study where cases were 1142 ESRD patients receiving transplantation and controls were 1184 kidney donors (a flowchart of the participants selection is shown in Figure 1). Second, to analyze whether *UMOD* affects long-term kidney transplant function, we performed a survival association analysis of donor rs12917707 genotype impact on incidence of GF in 1066 renal transplant recipients.

The *UMOD* gene expression product is uromodulin, also known as Tamm-Horsfall protein, which is excreted with urine, easily measurable [16-18] and thus presents a perfect intermediate phenotype for genetic association research. As the *UMOD* gene is expressed exclusively in the kidney, it was assumed that it was kidney genotype that was associated with urinary uromodulin in the previous reports [11-13,15]. To prove it, we aimed to investigate whether this association holds after the kidney is transplanted.

## Methods

### Study population

This study was conducted in the REGaTTA cohort [19,20]. Briefly, from all renal transplantations carried out in our center between 1993 and 2008 we included 1142 first graft recipients and 1186 donors (1066 matched donor-recipient pairs) for the present genetic study. The exclusion criteria were: re-transplantation, combined kidney/pancreas or kidney/liver transplantation, technical problems, absence of DNA and loss of follow-up. A flowchart of the study participants selection is shown in Figure 1. After transplantation the recipients were followed up for median [IQR] 5.5 [2.9–8.8] years and immunosuppression regimen, clinical and laboratory parameters, and time to GF were documented. GF was defined as return to dialysis or re-transplantation and was censored for death with a functioning graft. Patients characteristics, transplantation-related parameters, clinical and laboratory data were retrieved from medical records. The Institutional Review Board of the University Medical Center Groningen approved the study protocol. Informed consent was given by all transplant recipients and living donors. For deceased donors, with research carried out after the organ removal and implantation, no consent was required. According to Dutch law general consent for organ donation and transplantation includes consent for research projects. The study was conducted according to the principles of the Declaration of Helsinki. All the genetic and clinical data were anonymized prior to analyses.

### DNA isolation and genotyping

DNA was extracted from peripheral whole blood (in recipients and living donors) or lymph nodes/spleen lymphocytes (in deceased donors) using a commercial kit following the manufacturer’s instructions, transferred into 2 ml Eppendorf tubes and stored at -20°C. Absorbance at 260 nm was measured with NanoDrop spectrophotometer (ND-1000, NanoDrop Technologies) and DNA concentration was calculated by the NanoDrop nucleic acid application module. As a measure of DNA purity 260/280 and 260/230 absorbance ratios were assessed. Where samples failed to meet the minimum DNA concentration and purity recommended for Illumina genotyping, repeated isolation attempts were made. Genotyping of the rs12917707 SNP in the *UMOD* locus was performed using the Illumina VeraCode GoldenGate assay kit (Illumina, San Diego, CA, USA), according to the manufacturer’s instructions. Genotype clustering and calling were performed using BeadStudio Software (Illumina). Genotyping was unsuccessful in two individuals.

### Uromodulin measurement

For 282 outpatient renal transplant recipients at median [IQR] 4.2 [2.2-6.1] years post-transplant, 24 h urine samples were available at storage. Uromodulin concentration was measured by a commercially available enzyme-linked immunosorbent assay kit (MD Bioproducts, St. Paul, MN, Tamm-Horsfall Glycoprotein ELISA, catalog number M036020) according to the manufacturer’s instructions. For this assay, the interassay coefficient of variation is 10.5% at a mean concentration of 21.8 ng/mL and 12.2% at 95.1 ng/mL; the sensitivity is less than 0.75 ng/mL. The principle of the assay is based on a colorimetric sandwich immunoassay utilizing a polyclonal antibody against human uromodulin as the capture antibody and a biotinylated polyclonal antibody against human uromodulin as the detection antibody. Immediately after the ELISA procedure absorbance reading was performed at 450 nm. A standard curve was generated with each set of samples by reducing the data using 4-parameter logistic curve fit. Uromodulin concentration was calculated from the standard curve.

### Statistical analysis

Analyses were performed with PASW Statistics 18.0 (SPSS Inc., Chicago, IL) and PLINK v1.07 (S. Purcell, http://pngu.mgh.harvard.edu/purcell/plink/[21]). QUANTO v1.2.4 (http://hydra.usc.edu/gxe/) and PASS v11 were used for power estimation.

Deviation from Hardy-Weinberg equilibrium was tested in donors. Two individuals (donors), in which genotyping of rs12917707 was unsuccessful, were excluded and subsequent statistical analyses were performed on a final sample of 2326 subjects in a case-control design (1142 recipients vs 1184 donors) and 1066 matched donor-recipient pairs in a longitudinal design.

Genotype-phenotype associations were tested under an additive genetic model and results (regression coefficients and *p*-values) are reported per copy of the minor allele.

Since we tested the hypothesis that the minor rs12917707 allele is associated with reduced risk, such *a priori* directional prediction allowed us to assume statistical significance at a one-sided *p* = 0.05. Also in the reference paper [14] one-sided *p* values were presented.

With one-sided *p* = 0.05, assuming an additive genetic model and MAF of 20%, we had approximately 90% and 40% power to detect an OR of 0.8 and 0.9, respectively, in the ESRD case-control analysis, and 47% and 18% power to detect a HR of 0.8 and 0.9, respectively, in the longitudinal analysis of graft survival.

As in 164 (14%) cases transplantation was performed from living blood-related donors, the PLINK DFAM algorithm was used to account for relatedness in the case-control analysis.

We performed a fixed effects inverse variance meta-analysis to combine the results of our case-control study and the previously published one [14].

The effect of genotype on graft survival was investigated with Cox regression analysis including as covariates known predictors of GF (donor and recipient age and sex, donor type, ischemia times, delayed graft function and acute rejection episodes history, immunosuppression regimen).

To study association between genotype and uromodulin urinary levels after transplantation, due to small number of individuals homozygote for the minor allele, genotypes were combined into two groups: minor allele carriers (heterozygotes and homozygotes for the minor allele) and non-carriers (homozygotes for the ancestral allele). Statistical significance of differences between the groups was tested with a Mann-Whitney U test.

## Results

**Figure 2:**
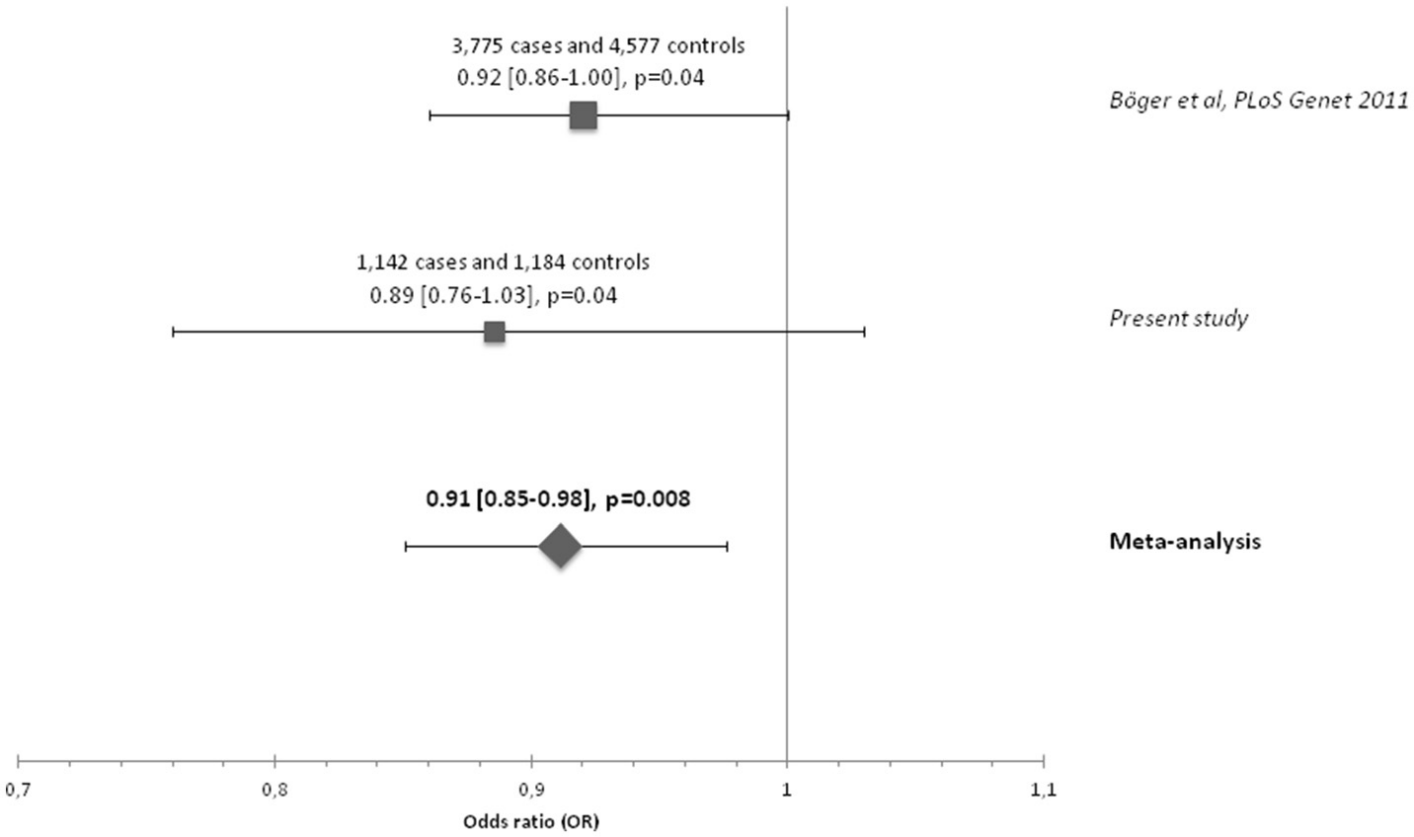
Meta-analysis forest plot.

Patient characteristics are presented in Table 1. There was no deviation from Hardy-Weinberg equilibrium in controls (*p* = 0.49). The rs12917707 minor allele frequency (MAF) in the overall sample was 17.3%, comparable to HapMap data and previous publications [7,8,14]. The MAF was 18.2% and 16.5% in kidney donors and ESRD patients, respectively. In the additive genetic model, adjusted for age, sex and case-control relatedness, OR [95% CI] for ESRD was 0.89 [0.76-1.03] per copy of the minor allele, one-sided *p* = 0.04, which is direction-consistent with the previously published association results [14]*.* A meta-analysis of our results and those of the abovementioned study [14] showed a significant association of rs12917707 with ESRD: OR [95% CI] 0.91 [0.85-98], *p* = 0.008 (Figure 2). There was no interaction between rs12917707 and age or sex (*p* = 0.92 and *p* = 0.97, respectively). We did not observe association between rs12917707 genotype and any of the underlying etiology of ESRD (Additional file 1: Table S1).

**Table 1:** Patient characteristics

**ESRD patients, n = 1142**	
Age, years	47.7 ± 13.5
Sex: male, n (%)	662 (58.0)
Primary disease:	
- glomerulonephritis, n (%)	242 (21.2)
- autosomal dominant polycystic kidney disease, n (%)	155 (13.6)
- pyelonephritis, n (%)	128 (11.2)
- renal vascular disease, n (%)	110 (9.6)
- IgA nephropathy, n (%)	89 (7.8)
- diabetes types I and II, n (%)	47 (4.1)
- other/uncertain etiology, n (%)	371 (32.5)
**Kidney donors, n = 1184**	
Age, years	44.5 ± 14.3
Sex: male, n (%)	602 (50.8)
Living donors, n (%)	282 (23.8)
- from which related donors, n (%)	164 (58.2)
**Matched donor-recipient pairs, n = 1066**	
Recipient age, years	48.1 ± 13.5
Recipient sex: male, n (%)	620 (58.2)
Donor age, years	44.6 ± 14.3
Donor sex: male, n (%)	540 (50.7)
Living donors, n (%)	261 (24.5)
Cold ischemia time, hours	17.4 [9.0-23.0]
Total warm ischemia time, minutes	40.0 [34.0-51.0]
Delayed graft function, n (%)	332 (31.1)
Post-transplant follow-up duration, years	5.5 [3.0-8.7]
Acute organ rejection episodes history, n (%)	368 (34.5)
Death-censored graft failure, n (%)	172 (16.1)
Death with a functioning graft, n (%)	182 (17.1)
**Renal transplant recipients with urine available, n = 282**	
Age, years	52.1 ± 12.3
Sex: male, n (%)	152 (53.9)
Urine collection time point, years after transplantation	4.2 [2.2-6.1]

Continuous normally distributed variables are presented as means ± SD, non-normally distributed – as medians [IQR].

During a median [IQR] 5.5 [2.9-8.8] years of follow-up, 172 (16.1%) cases of death-censored GF occurred and 183 (17.1%) patients died with a functioning graft. Donor kidney rs12917707 MAF was higher in subjects with a functioning graft as compared to cases that suffered death-censored GF (18.7% vs 17.2%, respectively). There was no significant association between donor rs12917707 and GF as in a univariate Cox regression analysis hazard ratio (HR) [95% CI] for GF was 0.92 [0.69-1.23], *p* = 0.57. A multivariate Cox regression model adjusted for known predictors of graft survival (Additional file 1: Table S2) showed a HR [95% CI] 0.96 [0.72-1.28], *p* = 0.76. Exclusion of cases with ≤1 year graft survival use did not change the results. Recipient rs12917707 also was not associated with GF (HR [95% CI] 1.04 [0.78-1.38], *p* = 0.788).

In a subset of renal transplant recipients (n = 282), in which 24-h urine collected at median [IQR] 4.2 [2.2-6.1] years post-transplant was available for uromodulin measurements, donor rs12917707 genotypes distribution was 4 / 94 / 184, correspondingly, MAF was 18.0%. Uromodulin concentrations ranged from 0.3 to 42.6 μg/ml, median [IQR] 8.6 [5.8-13.3] μg/ml, and were significantly lower in carriers of the rs12917707 minor allele in the donor genotype as compared to non-carriers: 7.3 [5.4-10.8] and 9.4 [6.4-15-6] μg/ml, respectively (*p* = 0.007) [Additional file 1: Figure S1]. Recipient rs12917707 was not associated with uromodulin urinary levels (*p* = 0.43).

## Discussion and conclusion

Thus, we have independently confirmed the association between *UMOD* rs12917707 and ESRD in a large case-control study. It was of similar direction and magnitude as previously reported [14], and in a meta-analysis of our results and the cited data convincing statistical significance was reached.

We did not find an association between rs12917707 genotype and etiology of ESRD. This suggests a universal, non-specific effect of the SNP on renal function decline irrespectively of underlying primary disease. A previous study described interaction between *UMOD* variant rs4293393 (in perfect LD with rs12917707) and age [12]. We, however, did not observe interaction with age or sex in our population.

Importantly, the observed relationship of the *UMOD* variant with ESRD in native kidneys did not translate into association with renal function loss in the transplanted kidney as we did not find an association between the donor rs12917707 and GF. Although the SNP effect on GF was direction-consistent with the case-control analysis and suggestive of a protective trend, the results were not statistically significant. It might indicate true absence of an association and differential involvement of *UMOD* in the pathophysiology of native and transplanted kidneys, or point to the fact that our longitudinal study was underpowered to detect the genetic effect due to the moderate sample size.

Remarkably, uromodulin urinary levels in patients after renal transplantation were associated with donor *UMOD* rs12917707 genotype. The subset of transplant recipients, in which urinary uromodulin was studied, was representative of the whole sample in terms of MAF and genotypes distribution. Uromodulin concentrations were significantly lower in recipient-carriers of the donor rs12917707 minor allele as compared to non-carriers. Thus, the genetic effect on uromodulin urinary level that was previously found in the native kidneys was reproduced in the transplanted kidneys, with similar direction of effect. This implies that it is indeed the *UMOD* genotype of the kidney that associates with uromodulin production.

Several arguments support the genetic analysis of a kidney transplant cohort. First, a case-control study with kidney donors, instead of the general population, as controls may have augmented statistical power to reveal the subtle genetic effects expected from common variants. Second, a transplant population provides the opportunity to study renal function loss in both native and transplanted kidneys through investigation of ESRD before and GF after transplantation. Finally, uniqueness and elegance of a transplantation setting is that it enables to test effects of both recipient and donor genotype on phenotype and thus discriminate between local (intra-renal) and systemic (extra-renal) processes.

The strengths of our study include the cohort’s size, its wide spectrum of underlying primary kidney disease and the specific design. However, some limitations deserve to be mentioned. Our longitudinal study may have been underpowered to detect significant SNP effect on GF. Power limitations also exist for the analysis of specific ESRD etiologies. Further, the analysis of the risk for ESRD is cross-sectional and needs to be confirmed by longitudinal studies studying incident ESRD. Unfortunately, the design and performance of such studies in CKD patients is challenging, and thus these are only emerging [22-24]. We did not have information on patients ethnicity, however, a reliable estimate for an average patient population in our region is that over 90% of the individuals are of European ancestry. Subsequently, our results are not generalizable to other ethnicities. Urinary uromodulin in renal transplant recipients was measured at different time points ranging from 1 to 9 years after transplantation. For those patients with presence of residual native kidney function, we cannot exclude a possible confounding effect of recipient rs12917707 genotype on urinary uromodulin concentration. However, this would have biased results to a null effect, while we have detected a significant association.

In summary, we have independently confirmed the association between genetic variation at the *UMOD* locus and ESRD. Also, donor kidney genotype was significantly associated with urinary uromodulin concentration in renal transplant recipients providing evidence that genetic make-up of the kidney determines this intermediate phenotype. Further research, including targeted sequencing of the region, bioinformatic analyses and functional experiments, is required to unravel the mechanisms by which common genetic variation at *UMOD* cause kidney disease.

## Competing interests

The authors of this manuscript have no competing interest.

## Authors’ contributions

AR, HS, MS and GN conceived and designed the experiments; AR, CAB, HS and GN analyzed the data; JvdB, MHdeB, JD, MCRFvD, HvG, BGH, JLH, HGDL, JN and SJLB contributed reagents/materials/analysis tools; AR, CAB, HS and GN wrote the manuscript. All authors read and approved the final manuscript.

## Pre-publication history

The pre-publication history for this paper can be accessed here:

http://www.biomedcentral.com/1471-2350/13/78/prepub

## Supplementary Material

Additional file 1 **Table S1.** Distribution of primary diseases in relation to *UMOD* rs12917707 genotype in ESRD patients. **Table S2.** Predictors of GF (univariate Cox regression analysis) in 1066 renal transplant recipients followed up for a median [IQR] 5.5 [2.9-8.8] years after transplantation. **Figure S1.** Uromodulin urinary concentration in 282 renal transplant recipients at 4.2 [2.2-6.1] yrs post-transplant by donor and recipient genotype stratified by presence (carriers) or absence (non-carriers) of *UMOD* rs12917707 minor allele. Click here for file
