# Supplementary material for: UMOD as a susceptibility gene for end-stage renal disease
Source: BMC Med Genet. 2012 Sep 5;13:78. doi: 10.1186/1471-2350-13-78 (PMC3495046; doi:10.1186/1471-2350-13-78)
Supplement: Additional file 1 — Table S1. Distribution of primary diseases in relation to UMOD rs12917707 genotype in ESRD patients. Table S2. Predictors of GF (univariate Cox regression analysis) in 1066 renal transplant recipients followed up for a median [IQR] 5.5 [2.9-8.8] years after transplantation. Figure S1. Uromodulin urinary concentration in 282 renal transplant recipients at 4.2 [2.2-6.1] yrs post-transplant by donor and recipient genotype stratified by presence (carriers) or absence (non-carriers) of UMOD rs12917707 minor allele. [file 1471-2350-13-78-S1.pdf]

Supplementary Material

Table S1. Distribution of primary diseases in relation to *UMOD* rs12917707 genotype in ESRD patients.

	***UMOD* rs12917707**			*P* value
	**0**	**1**	**2**	
N	799	310	33	
Glomerulonephritis, n (%)	168 (21.0)	69 (22.3)	5 (15.2)	0.623
Polycystic kidney disease, autosomal dominant (adult) form, n (%)	111 (13.9)	37 (11.9)	7 (21.2)	0.298
Pyelonephritis, n (%)	91 (11.4)	35 (11.3)	4 (12.1)	0.990
Renal vascular disease, n (%)	81 (10.1)	26 (8.4)	3 (9.1)	0.671
IgA nephropathy, n (%)	61 (7.6)	26 (8.4)	2 (6.1)	0.853
Diabetes type I and II, n (%)	33 (4.1)	14 (4.5)	0 (0.0)	0.462

Digits 0-2 designate number of the rs12917707 minor allele copies per genotype

Table S2. Predictors of GF (univariate Cox regression analysis) in 1066 renal transplant recipients followed up for a median [IQR] 5.5 [2.9-8.8] years after transplantation

	HR	95% CI	*P* value
Recipient age at transplantation	0.99	0.98-1.00	0.037
Recipient sex: male	1.11	0.82-1.51	0.495
Donor age	1.02	1.01-1.03	0.002
Donor sex: male	0.98	0.73-1.33	0.907
Donor type: living vs deceased	0.54	0.35-0.84	0.006
Cold ischemia time	1.03	1.01-1.04	0.001
Total warm ischemia time	1.02	1.01-1.03	0.001
Delayed graft function	3.83	2.83-5.19	<0.001
Acute rejection episodes history	1.87	1.38-2.52	<0.001

HR, hazard ratio; CI, confidence intervals.

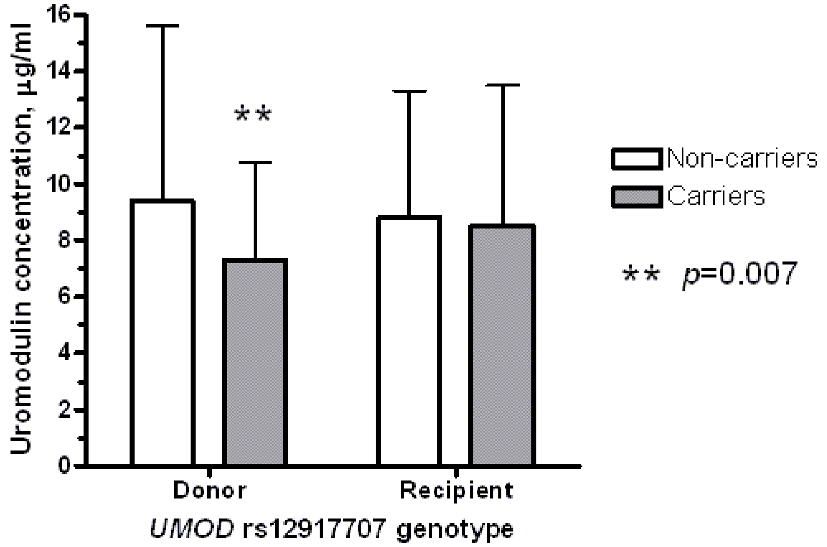

Figure S1. Uromodulin urinary concentration in 282 renal transplant recipients at 4.2 [2.2-6.1] yrs post-transplant by donor and recipient genotype stratified by presence (carriers) or absence (non-carriers) of *UMOD* rs12917707 minor allele. Columns represent respective medians of values, error bars – interquartile range. *P*=0.007, Mann-Whitney U-test.
